# Subchronic Microcystin-LR Aggravates Colorectal Inflammatory Response and Barrier Disruption via Raf/ERK Signaling Pathway in Obese Mice

**DOI:** 10.3390/toxins15040262

**Published:** 2023-04-01

**Authors:** Yue Yang, Shuilin Zheng, Hanyu Chu, Can Du, Mengshi Chen, Mohammed Y. Emran, Jihua Chen, Fei Yang, Li Tian

**Affiliations:** 1 Hunan Provincial Key Laboratory of Clinical Epidemiology, Xiangya School of Public Health, Central South University, Changsha 410078, China; 2 Hunan Province Key Laboratory of Typical Environmental Pollution and Health Hazards, The Key Laboratory of Ecological Environment and Critical Human Diseases Prevention of Hunan Province, Department of Education, Hengyang Medical School, University of South China, Hengyang 421001, China; 3 Changsha Center for Disease Control and Prevention, Changsha 410004, China; 4 National Institute for Materials Science (NIMS), 1-2-1 Sengen, Tsukuba 305-0047, Japan; 5 Department of Gastroenterology, Third Xiangya Hospital, Central South University, Changsha 410013, China

**Keywords:** microcystin-LR, colorectal injury, subchronic toxicity, high-fat diet, inflammation responses

## Abstract

Microcystin-LR (MC-LR) is an extremely poisonous cyanotoxin that poses a threat to ecosystems and human health. MC-LR has been reported as an enterotoxin. The objective of this study was to determine the effect and the mechanism of subchronic MC-LR toxicity on preexisting diet-induced colorectal damage. C57BL/6J mice were given either a regular diet or a high-fat diet (HFD) for 8 weeks. After 8 weeks of feeding, animals were supplied with vehicle or 120 μg/L MC-LR via drinking water for another 8 weeks, and their colorectal were stained with H&E to detect microstructural alterations. Compared with the CT group, the HFD and MC-LR + HFD-treatment group induced a significant weight gain in the mice. Histopathological findings showed that the HFD- and MC-LR + HFD-treatment groups caused epithelial barrier disruption and infiltration of inflammatory cells. The HFD- and MC-LR + HFD-treatment groups raised the levels of inflammation mediator factors and decreased the expression of tight junction-related factors compared to the CT group. The expression levels of p-Raf/Raf and p-ERK/ERK in the HFD- and MC-LR + HFD-treatment groups were significantly increased compared with the CT group. Additionally, treated with MC-LR + HFD, the colorectal injury was further aggravated compared with the HFD-treatment group. These findings suggest that by stimulating the Raf/ERK signaling pathway, MC-LR may cause colorectal inflammation and barrier disruption. This study suggests that MC-LR treatment may exacerbate the colorectal toxicity caused by an HFD. These findings offer unique insights into the consequences and harmful mechanisms of MC-LR and provide strategies for preventing and treating intestinal disorders.

## 1. Introduction

Under the combined effects of global water eutrophication and climatic warming, cyanobacterial blooms have sparked considerable public concern, and the cyanobacterial toxins emitted by cyanobacterial cells pose a grave danger to animal and human health [1,2,3]. Microcystins (MCs) are secondary metabolites produced by cyanobacteria like *Microcystis* and *Anabaena* during epidemics of algal blooms and are among the most widely dispersed cyanobacterial toxins in freshwater [4,5]. So far, more than 270 varieties of MCs have been documented [6]. Microcystin-LR (MC-LR), microcystin-RR (MC-RR), and microcystin-YR (MC-YR) are the most extensively dispersed variants in nature, with MC-LR being the most widely distributed and the most dangerous [7]. Many organs, including the liver, intestines, kidneys, nerves, immunological system, and reproductive system, are harmed by MC-LR when it enters the body via consumption of water or freshwater products [8,9,10,11,12,13]. In 2021, a study reported the abrupt death of 330 African elephants due to algal toxins (concentrations of up to 1.2 × 10^5^ μg/L) in continental African waters, prompting fear and alarm on a global scale [14]. MC-LR contamination poses a grave hazard to the safety of drinking water and human health. The World Health Organization (WHO) has defined the tolerated daily intake (TDI) for MC-LR at 0.04 μg/(kg·d bw), and the safety threshold of MC-LR in drinking water for residents is 1 μg/L [15].

The liver is the primary target organ for MC-LR, but the harm to other organs should not be overlooked [10]. In addition to liver damage, several studies indicate that MC-LR can also cause damage to the gastrointestinal tract of animals and humans [6,11,16]. A cross-sectional epidemiological investigation revealed that direct exposure with MC-LR can cause intestinal symptoms such as nausea, vomiting, abdominal discomfort, and diarrhea and suggested that MC-LR concentration may be positively linked with the incidence of colorectal cancer [17]. According to the findings of our earlier investigation, MC-LR exposure can also induce inflammatory responses in colorectal and jejunal tissues [6,16]. Obesity, diabetes, and hypertension have been connected with intestinal damage (including inflammatory bowel disease) [18,19]. It is believed that exposure to environmental contaminants contributes to both intestinal disease and an increase in the prevalence of obesity-induced nonalcoholic fatty liver disease (NAFLD). Previous research has demonstrated that MC-LR exposure exacerbates the NAFLD and nephrotoxicity generated by a high-fat, high-cholesterol (HFHC) diet [20,21]. Most of the current research on MC-LR, including studies to calculate current MC-LR TDI values, has been undertaken on healthy animals [22]. However, there is a growing desire to identify people at risk who may be more sensitive to MC-LR-induced enterotoxicity. Given the tight relationship between obesity and intestinal disorders, we expected that MC-LR would also worsen obesity-induced intestinal toxicity.

Extracellular signal-regulated kinase (ERK) is a member of the mitogen-activated protein kinases (MAPK) family of mitogen-activated protein kinases [23]. Current studies suggest that the ERK pathway significantly promotes intestinal epithelial cell differentiation and proliferation and inhibits intestinal epithelial cell apoptosis [24]. Song et al. showed that, p-ERK/ERK and proinflammatory factors (IL-1β, IL-6, TNF-α, and MPO) expression was increased in patients with ulcerative colitis (UC), and inhibition of ERK expression significantly inhibited the occurrence of dextran sulphate sodium (DSS)-induced inflammatory responses in wild-type (WT) mice [25]. Wei et al. showed that ERK can induce Ras/Raf cascade activation and activate the Wnt/β-catenin pathway, affecting intestinal epithelial cell proliferation and migration [26]. Cai et al. showed that Keratinocyte growth factor (KGF) can regulate E-cadherin expression in intestinal epithelial cells through activation of the ERK signaling pathway and affect apoptosis [27]. However, no studies have yet explored the role and mechanism of ERK-related signaling pathways in MC-LR and HFD-induced intestinal injury.

Therefore, it was proposed that the present investigation investigate for the first time whether subchronic MC-LR exposure could increase pre-existing diet-induced intestinal injury in mice and its related mechanisms by establishing a model of cotreatment with MC-LR (120 g/L via drinking water for 8 weeks) and a high-fat diet. This research may provide insights into, and new possibilities for clarifying, the intestinal toxicity of MC-LR and its relationship with high-fat diet-induced damage.

## 2. Results

### 2.1. Impact of MC-LR Treatment on the General Condition and Body Weight of Obese Mice

We use the same animal model as that from our previous study and the results are consistent with this study [28]. For the duration of the exposure, the body weights of the mice were measured every 2 weeks. During the 16-week experiment, neither mouse deaths nor abnormal behavior was found. As shown in Table 1, the weight of the high-fat diet (HFD)-treatment group increased significantly more than the control group (CT) after 16 weeks. However, the MC-LR + HFD-treatment group showed no effect on the HFD-induced increase in weight gain.

### 2.2. Impact of MC-LR Treatment on the Histopathological Changes in the Colorectal Tissue of Obese Mice

**Figure 1:**
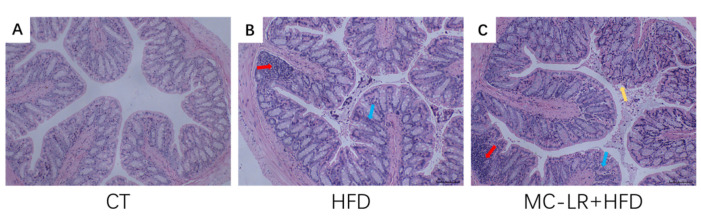
Analysis of the histopathology of colorectal tissues from mice in multiple treatment groups. (**A**) CT-treatment group; (**B**) HFD-treatment group; and (**C**) MC-LR + HFD-treatment group. The red arrow represents lymphocyte infiltration, the yellow arrow represents tissue mucosal shedding, and the blue arrow represents a disordered crypt architecture. Bar = 100 μm means original magnification × 100.

Using Hematoxylin-Eosin (HE)-stained tissue sections from each mouse, we detected the pathological alterations of the colorectal tissue (Figure 1). Compared to the CT group, the HFD-treatment group exhibited abnormal intestinal crypt architecture and epithelial organization in the crypt, as well as inflammatory cell infiltration. In addition, MC-LR treatment exacerbated the colorectal damage induced by HFD treatment. In obese mice, MC-LR considerably exacerbated the inflammatory response in the colorectum.

### 2.3. Impact of MC-LR Treatment on the Expression of Inflammatory Mediators Factors in the Colorectal Tissue of Obese Mice 

**Figure 2:**
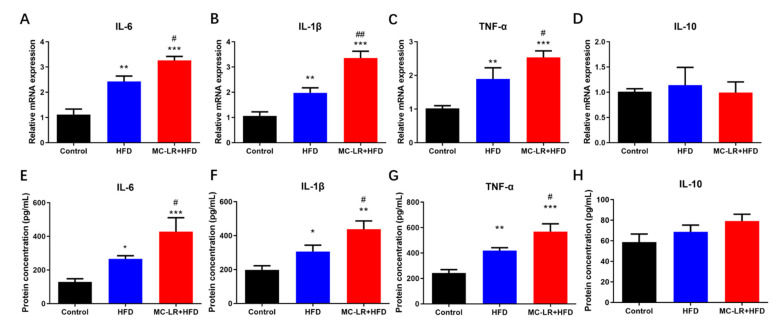
Impact of MC-LR on the expression levels of inflammatory factors in the colorectal of HFD-induced obese mice. (**A**) The mRNA levels of IL-6; (**B**) The mRNA levels of IL-1β; (**C**) The mRNA levels of TNF-α; (**D**) The mRNA levels of IL-10; (**E**) The protein concentration of IL-6; (**F**) The protein concentration of IL-1β; (**G**) The protein concentration of TNF-α; and (**H**) The protein concentration of IL-10. Results are presented as the mean ± SD; *n* = 5. * *p* < 0.05, ** *p* < 0.01, *** *p* < 0.001 vs. CT group; # *p* < 0.05, ## *p* < 0.01 vs. with HFD-treatment group.

The expression levels of inflammatory markers IL-6, IL-1β, TNF-α, and IL-10 were evaluated by quantitative real-time PCR (qRT-PCR) and enzyme-linked immunosorbent assay (ELISA) to determine if MC-LR exacerbates colorectal inflammation in obese mice caused by an HFD. The results are illustrated in Figure 2. The expression of inflammatory factors (IL-6, IL-1, TNF-, and IL-10) was elevated in the HFD -and MC-LR + HFD-treatment groups compared to the CT group. In addition, these proinflammatory variables were all significantly elevated in the MC-LR + HFD-treatment group compared to the HFD-treatment group.

### 2.4. Impact of MC-LR Treatment on the Expression of Tight Junction-Related Factors in the Colorectal Tissue of Obese Mice 

**Figure 3:**
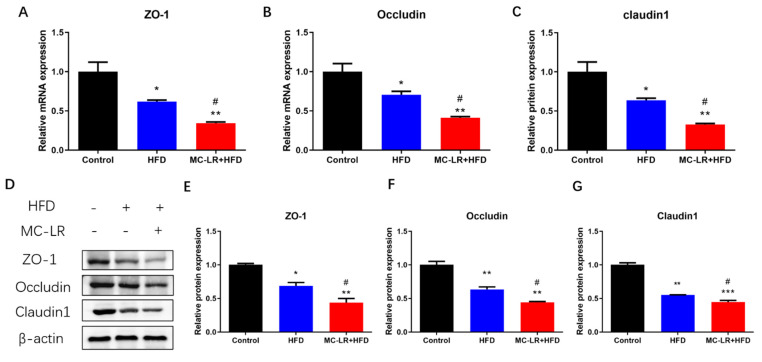
Impact of MC-LR on tight junction-related factors expression levels in the colorectum of HFD-induced obese mice. (**A**) The mRNA levels of ZO-1; (**B**) The mRNA levels of Occludin; (**C**) The mRNA levels of Claudin1; (**D**) Relative protein levels are shown normalized to β-actin; (**E**) The protein levels of ZO-1; (**F**) The protein levels of Occludin; and (**G**) The protein levels of Claudin1. Results are presented as the mean ± SD; *n* = 5. * *p* < 0.05, ** *p* < 0.01, *** *p* < 0.001 vs. CT group; # *p* < 0.05 vs. HFD-treatment group.

To evaluate if MC-LR exacerbates colorectal barrier disruption, the mRNA levels of tight junction-related factors ZO-1, Occludin, and Claudin1 were measured by qRT-PCR. Figure 3A–C illustrates the outcomes. The relative levels of mRNA for ZO-1, Occludin, and Claudin1 were considerably lower in the HFD-treatment group compared to the CT group, a trend that was worsened by MC-LR treatment.

In addition, western blotting (WB) was used to examine changes in the protein levels of these tight junction-related factors. Figure 3D–G demonstrates the outcomes. In comparison to the CT group, all tight junction-related factors were significantly downregulated in the HFD- and MC-LR + HFD-treatment groups. In addition, these tight junction-related factors were all significantly decreased in the MC-LR + HFD-treatment compared to the HFD-treatment group.

### 2.5. Impact of MC-LR Treatment on the Expression of Raf/ERK Signaling Pathway-Related Proteins in the Colorectal of Obese Mice

**Figure 4:**
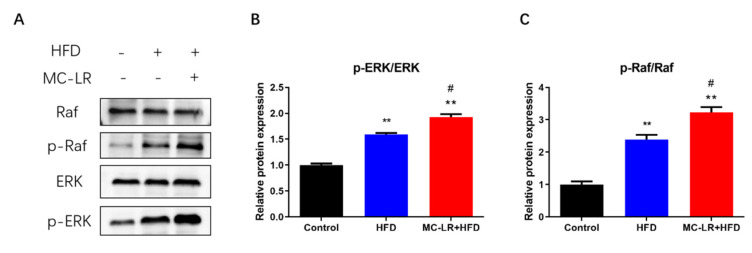
Impact of MC-LR on Raf/ERK pathway in the colorectum of HFD-induced obese mice. (**A**) WB analysis of proteins (p-Raf, Raf, p-ERK, and ERK). (**B**) Relative quantitation of p-ERK/ERK protein level normalized to β-actin. (**C**) Relative quantitation of p-Raf/Raf protein level normalized to β-actin. Results are presented as the mean ± SD; *n* = 5. ** *p* < 0.01 vs. CT group; # *p* < 0.05 vs. HFD-treatment group.

To further investigate the biochemical pathway by which MC-LR exacerbates colorectal damage in obese mice fed an HFD, the levels of p-Raf, Raf, p-ERK, and ERK were measured by WB. As shown in Figure 4, the expression of p-Raf/Raf and p-ERK/ERK were considerably higher in the HFD- and MC-LR + HFD-treatment groups compared to the CT group; the expression levels of these proteins were also significantly higher in the MC-LR + HFD-treatment group compared to the HFD-treatment group. By activating the Raf/ERK signaling pathway, MC-LR exacerbates colorectal damage in obese mice fed an HFD.

## 3. Discussion

### 3.1. MC-LR Promoted Colorectal Damage in Obese Mice

Throughout the experiment, the body weights of the mice were observed. The primary study demonstrated that the weight of mice fed an HFD increased significantly more than that of mice on a standard diet. However, after treatment with MC-LR, there was no notable change in weight as compared to the HFD-treatment group. Similarly, Arman et al. reported the body weight effects of the MC-LR and HFHC diets [20]. Our observations are congruent with the results. MC-LR had no influence on either of the diet groups’ body weights.

The histopathological findings revealed an increase in overall colorectum pathology, inflammatory cell infiltration, and intestinal barrier damage in the HFD-treatment group following MC-LR exposure, indicating an increased susceptibility to MC-LR-stimulated colorectal damage in the context of a poor diet. Ferreira et al. discovered that after 15 days of exposure of white chub to MC-LR-containing water, the muscular layer of the intestine was injured, with considerable fibrous necrosis and severe necrosis of epithelial cells [29]. Chen et al. exposed zebrafish to 20 µg/L MC-LR for 30 days and observed a partial loss of intestinal villi, a change in villi length, and severe necrotizing enterocolitis [30]. Cao et al. stained mice with MC-LR drinking water for 6 months and discovered that the morphology of the mice’s jejunal tissue was dramatically altered, as was the arrangement of intestinal epithelial cells, and lymphocyte infiltration was seen [16]. Mice with colorectal tissues were able to develop considerably enhanced lymphocyte infiltration, altered crypt alignment, and intestinal barrier degradation after exposure to low concentrations of MC-LR for 12 months [6]. Using various staining techniques, it was discovered that MC-LR may produce acute and chronic colitis in different animal species. Su et al. also discovered that MC-LR extended blood stool duration, exacerbated colonic mucosa ulcer, and reduced colon length in mice with DSS-induced colitis [31]. For MC-LR-induced organ damage in a poor dietary environment, only the study by Arman et al. and Chu et al. found that MC-LR exposure causes more severe HFHC/HFD-induced NAFLD and kidney damage [20,21,28]. Our data indicate for the first time that MC-LR exposure increases colorectal damage in the HFD-treatment group. MC-LR may cause colorectal damage by triggering an inflammatory response.

### 3.2. MC-LR Promoted Colorectal Inflammatory Response and Barrier Disruption in Obese Mice

Consistent with our previous findings, the elevated expression of inflammation-related factors (IL-6, IL-1β, and TNF-α) in this study was caused by MC-LR [6]. Rocha et al. demonstrated that MC-LR induced the release of IL-1β and INF-α from peritoneal macrophages, indicating that MC-LR may activate certain biological pathways via IL-1β as well as INF, thereby affecting the electrophysiological secretion of the intestine and its physiological functions [32]. After 6 months of MC-LR drinking-water staining in mice, Cao et al. found increased expression of proinflammatory factors IL-1β, IL-8, and TNF-α mRNA in the jejunum, thereby exacerbating the inflammatory response in the jejunum [16]. Chen et al. exposed zebrafish to MC-LR-containing water for 30 days and revealed that the levels of inflammatory factors IFN-1, IL-1β, IL-8, and TNF-α were significantly elevated in their intestines, and that MC-LR increased proinflammatory factor gene expression-induced tissue damage and inflammatory responses in zebrafish [30]. Su et al. demonstrated that MC-LR significantly increased the expression of proinflammatory transcripts (TNF-, IL-1, CD40, and MCP-1) and profibrosis markers PAI-1 and MCP-1 in colon tissues of mice with colorectal inflammation induced by DSS, compared to DSS ingestion alone [31,33]. In conclusion, the imbalance between proinflammatory and anti-inflammatory factors is a crucial factor in MC-LR-induced intestinal inflammation. IL-6 not only promotes the chemotaxis of neutrophils to inflammatory lesions, but also activates the downstream RAS/MAPK pathway after specific binding to receptors in enterocytes, inducing apoptosis and accumulation of T cells, resulting in an increase in inflammatory factors and dysfunction in the intestinal mucosa [34]. IL-1β directly stimulates neutrophils, causing them to produce inflammatory proteins and inflammatory mediators to participate in the inflammatory process, which mediates neutrophil chemotaxis by stimulating monocytes and macrophages to secrete proinflammatory factors, including IL-6, IL-8, and TNF-α [35]. TNF-α binds to its receptors TNFR1 and TNFR2 and activates diverse signaling pathways to activate proinflammatory-related transcription factors and nuclear factor NF-κB, as well as to stimulate more TNF-α production by macrophages and dendritic cells via proinflammatory factors IL-1 and IL-6. TNF-α promotes the release of adhesion factors, such as ICAM-1 and VCAM-1, that leads to the accumulation of granulocytes and the amplification of the initial inflammatory response [36]. This work has demonstrated that MC-LR can exacerbate HFD-induced colorectal damage in obese mice by triggering an inflammatory response. 

Moreover, the levels of tight junction-related factors (ZO-1, Occludin, and Claudin1) were significantly reduced in our study, indicating that the intestinal barrier was disrupted. There are currently few studies on intestinal barrier damage caused by MC-LR treatment. An in vitro study revealed that MCs can affect intestinal barrier function by decreasing the expression of cytoskeletal proteins closely linked to Occludin and ZO-1 in IECs and by dose-dependently impairing the integrity of the intestinal barrier [37]. Zhuang et al. also discovered that chronic MC-LR contamination of drinking water in mice for 6 months decreased the expression of mRNA for intestinal tight junction Claudin, Occludion, and ZO-1 [38]. The mRNA levels of intestinal epithelial tight junction-related genes (Claudin-5, Occludin, and ZO-1) were significantly reduced in zebrafish reared for 21 days with MC-LR (35 μg/L) [39]. Several of the aforementioned studies support our findings that MC-LR exposure can cause intestinal barrier damage and increased intestinal permeability. Tight junctions are a crucial component of the intestinal barrier, which is composed of numerous tight junction proteins (Occludin protein family, Claudin protein family, cytoplasmic proteins of ZO-1, ZO-2, ZO-3, cingulin, etc.) [40]. In the absence of harmful factors, intestinal tight junctions function as a selective barrier to maintain barrier function and intestinal homeostasis. When the tight junctions are damaged or destroyed, the intestinal barrier is compromised, and intestinal permeability increases, harmful factors can enter the body and cause IBD, celiac disease, diabetes, and other conditions [41]. A population-based study revealed that patients with IBD had significantly decreased expression of intestinal epithelial tight junction-related proteins, increased intestinal permeability, severe impairment of barrier function, and a correlation between the severity of the inflammatory response to the disease and disruption of the tight junctions in the mucosa [42]. Experiments on animals demonstrated that DSS caused intestinal tight junction structure disruption, intestinal barrier damage, and elevated permeability in mice with colitis [43]. Sarkar et al. discovered a significant decrease in intestinal tight junction protein expression in nonalcoholic fatty liver mice stained with subchronic MC-LR [44]. In this investigation, we discovered that obese mice exposed to MC-LR had impaired tight junction proteins, intestinal barrier impairment, and increased intestinal permeability. Hence, the results of this research reveal for the first time that MC-LR could accelerate the development of severe colorectal disease in obese mice by decreasing the expression of tight junction-related factors.

### 3.3. MC-LR Promoted Colorectal Inflammatory Response and Barrier Disruption by Activating Raf/ERK Signaling Pathway in Obese Mice

In this research, exposure to MC-LR elevated p-Raf/Raf and p-ERK/ERK protein expression in the HFD-treatment group. It has been proven that MC-LR activates the Raf/ERK signaling pathway to promote colorectal damage in obese mice. The Raf/ERK signal-grade pathway is a widely activated MAPK pathway that can transmit extracellular signals into the nucleus, induce changes in the expression profile of heteroglyphic proteins, and regulate diverse cellular processes including cell proliferation, growth, differentiation, transformation, and apoptosis [23]. By managing the imbalance of several factors, the Raf/ERK signaling pathway can affect inflammation and possibly cancer [45,46]. Chen et al. found that chronic low-dose MC-LR exposure upregulated type 3 deiodinase expression and ultimately interfered with thyroid–hormone synthesis and metabolism by activating the p38/MAPK and MEK/ERK signaling pathways in mice exposed to the compound orally for 6 months [47]. Sun et al. demonstrated that in normal hepatocytes HL7702, MC-LR increased the phosphorylated expression of ERK, which increased the expression of E-cadherin and p-paxillin proteins and altered their colocalization, thereby decreasing the adhesion of hepatocytes, and that ERK protein inhibitors reversed this phenomenon [48]. Dong et al. also discovered that Ntrk1 promotes thylakoid cell proliferation and proinflammatory factor expression in MsPGN rats by activating the p38/ERK MAPK signaling pathway, thereby initiating inflammatory responses [49]. However, there is no research on the function of Raf/ERK signaling pathways in MC-LR-induced obese mice. In obese mice, we first found that MC-LR treatment significantly increased Raf/ERK signaling pathway activation, promoting colorectal inflammatory response and barrier damage. We propose that these pathways contribute to the aggravation of colorectal injury by MC-LR in obese mice.

## 4. Conclusions

This is the first study to show that exposure to MC-LR in the context of an HFD may dysregulate inflammatory mediators, damaging the intestinal barrier. The Raf/ERK signaling pathway was activated, inducing increased expression of inflammatory factors and suppressing tight junction factor expression, leading to a colorectal inflammatory response and barrier damage. Our research may yield unique insights for preventing obesity-related MC-LR environmental risk factors. However, additional research is required to determine whether obesity increases the likelihood of MC-LR-induced colorectal toxicity.

## 5. Materials and Methods

### 5.1. Chemicals and Reagents

MC-LR with a purity of 95% was purchased from Alexis Corporation (Lausen, Switzerland). RIPA buffer, bicinchoninic acid (BCA) protein assay kit was purchased from Beyotime (Shanghai, China). The polyvinylidene fluoride (PVDF) membrane was purchased from Merck Millipore Ltd., (Billerica, MA, USA). The phosphatase inhibitor cocktail and protease inhibitor cocktail were purchased from CWBIO (Beijing, China). Trizol Reagent was purchased from Invitrogen Life Technologies (Carlsbad, CA, USA). The HiScript^®^ II Q RT SuperMix for qRT-PCR and ChamQ Universal SYBR qRT-PCR Master Mix were purchased from Vazyme (Nanjing, China). The ERK antibody, p-ERK antibody, Raf antibody, p-Raf antibody, ZO-1 antibody, Occludin antibody, Claudin1 antibody, β-actin antibody, HRP-conjugated Affinipure Goat Anti-Rabbit IgG (H + L), and HRP-conjugated Affinipure Goat Anti-Mouse IgG (H + L) were purchased from Proteintech (Wuhan, China). 

### 5.2. Animals and Diet

Six- to eight-week-old male C57BL/6J mice were purchased from the Hunan SJA Laboratory Animal Co., Ltd. (Changsha, China) and maintained on a standard laboratory condition (22–24°C, 40–70% relative humidity, and a 12:12 h light-dark cycle). The control group (*n* = 5) consisted of mice fed a control diet (D12450J, Research Diets Inc., New Brunswick, NJ, USA), whereas the other mice (*n* = 20) were fed an HFD (D12492, Research Diets Inc., New Brunswick, NJ, USA) for 8 weeks to establish obesity models. The body weights of 7 mice did not exceed 20% of the CT group’s average weight. Thirteen obese mice whose body weight exceeded 20% of the average weight of the CT group were obtained after 8 weeks. Ten obese mice were further divided into 2 groups, including the HFD group (*n* = 5) and the MC-LR + HFD group (*n* = 5). All 3 groups of mice were fed with a control diet, an HFD and the HFD with 120 μg/L MC-LR in the drinking water, respectively, for another 8 weeks. No mice died during the process. Their weight was determined every 2 weeks. The Central South University Animal Care and Use Committee approved all animal experiments (Approval Number: XYGW-2018-41).

### 5.3. Histological Analysis

Immediate isolation and evaluation of colorectal tissue samples were conducted. Cold phosphate-buffered solution (PBS, pH7.2) was used to remove the bloodstains, and 4% paraformaldehyde (PFA) was used to preserve them overnight at room temperature. These tissues had been paraffin-embedded. After dewaxing, 4 m-thick slices of tissue were stained with HE. The sections were then observed and photographed using an optical microscope (Motic, BA210).

### 5.4. WB

The BCA technique was used to determine protein concentration. Using SDS-PAGE, proteins were separated and electroblotted upon a PVDF membrane. Protein Free Rapid Blocking Buffer was used to block the transferred membrane. Antibodies were treated with membranes overnight at 4°C. Sixty min were spent incubating the transplanted membrane with goat anti-mouse IgG (H + L) HRP conjugate or goat anti-rabbit IgG (H + L) HRP conjugate. Protein bands were detected using Luminata Forte Western HRP substrate and quantified using a Bio-Rad chemiluminescence imaging system (Bio-Rad, Hercules, CA USA). ImageJ was utilized to quantify the band’s intensity (Rawak Software, Inc., Stuttgart, Germany).

### 5.5. qRT-PCR

RNA was isolated from colorectal tissues using the Trizol Reagent. RNA was reverse-transcribed using the HiScript^®^ II Q RT SuperMix for qRT-PCR. On a qTOWER3 Real-Time PCR System, qRT-PCR was conducted with ChamQ Universal SYBR qRT-PCR Master Mix (Analytikjena, Germany). β-actin was utilized to normalize mRNA expression. The relative amounts of mRNA were assessed using the 2^−*ΔΔCt*^ method. Each experiment was conducted in triplicate. Primer Premier 6.0 was used to design the primers, which are listed in Table 2.

### 5.6. ELISA

Using the ELISA double-antibody sandwich approach (Neobioscience, Shenzhen, China), the levels of the inflammatory cytokines IL-1β, IL-6, TNF-α, and IL-10 were determined. The assay procedure strictly followed the kit’s instructions. Using an automatic enzyme microplate (BioTek, Winooski, VT, USA), the absorbance at 450 nm was determined.

### 5.7. Statistical Analysis

Each experiment was performed a least 3 times per modality. The data wereprovided as the mean ± SD, and comparisons were considered statistically significant when *p* < 0.05. A 1-way ANOVA was conducted using SPSS version 22.0 (SPSS Inc., Chicago, IL, USA) to assess the statistical differences between the treatment groups.

## Figures and Tables

**Table 1:** Effects of MC-LR on body weight in HFD-induced obese mice ($\bar{\chi }$ ± SD, *n* = 5).

Group	Body Weight (g)
Control	33.96 ± 1.374
HFD	50.58 ± 2.312 ***
HFD + MC-LR	51.52 ± 2.782 ***

Note: *** *p* < 0.001 compared with CT group.

**Table 2:** Primer sequences for qRT-PCR.

Genes	Forward Primer (5′–3′)	Reverse Primer (5′–3′)
*IL-6*	CCACGGCCTTCCCTACTTC	TTGGGAGTGGTATCCTCTGTGA
*TNF-α*	CCCACGTCGTAGCAAACCA	ACAAGGTACAACCCATCGGC
*IL-1β*	GCACTACAGGCTCCGAGATGAA	GTCGTTGCTTGGTTCTCCTTGT
*IL-10*	AGAGCTGCGGACTGCCTTCA	ACCTGCTCCACTGCCTTGCT
*ZO-1*	GCGATTCAGCAGCAACAGAACC	AGGACCGTGTAATGGCAGACTC
*Occludin*	GCGGCTATGGAGGCTATGGCTA	AGGAAGCGATGAAGCAGAAGGC
*Claudin1*	GGACAACATCGTGACCGCTCAG	TCCAGGCACCTCATGCACTTCA
*β-Actin*	TCAAGATCATTGCTCCTCCTGAG	ACATCTGCTGGAAGGTGGACA

## Data Availability

The data presented in this study are available in this article.
